# Synthesis of phosphorothioates using thiophosphate salts

**DOI:** 10.1186/1860-5397-2-4

**Published:** 2006-03-16

**Authors:** Babak Kaboudin, Fatemeh Farjadian

**Affiliations:** 1 Department of Chemsitry, Institute for Advanced Studies in Basic Sciences, Gava Zang, Zanjan 45195-1159, Iran

## Abstract

Reactions of *O,O*'-dialkyl thiophosphoric acids with alkyl halides, in the presence of a base, provide a direct synthetic route to phosphorothioates via *O,O*'-dialkyl thiophosphate anion formation. Studies on the reaction of ambident nucleophile ammonium *O,O*'-diethyl thiophosphate with benzyl halides and tosylate in different solvents show that only *S*-alkylation is obtained. Reaction of this ambident nucleophile with benzoyl chloride (a hard electrophile), gave the *O*-acylation product. A simple, efficient, and general method has been developed for the synthesis of phosphorothioates through a one-pot reaction of alkyl halides with the mixture of diethyl phosphite in the presence of triethylamine/sulfur/and acidic alumina under solvent-free conditions using microwave irradiation.

## Introduction

**Scheme 1:**
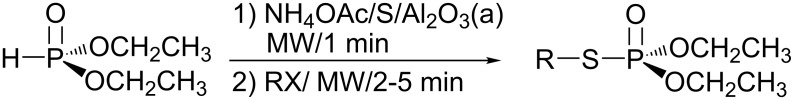
Synthesis of phosphorothioates using microwave irradiation

Organophosphorus compounds have found a wide range of application in the areas of industrial, agricultural, and medicinal chemistry owing to their biological and physical properties as well as their utility as synthetic intermediates. [1] The synthesis of phosphate esters is an important objective in organic synthesis, since they have found use in the preparation of biologically active molecules, and also versatile intermediate in synthesis of amides and esters. [2–4] Among the phosphate esters, phosphorothioate derivatives are of interest as effective pesticides. [5–8] In recent years a number of phosphorothioates have been introduced as potential chemotherapeutic agent. [9–12] Despite their wide range of pharmacological activity, industrial and synthetic applications, the synthesis of phosphorothioates has received little attention. The following methods, not generally applicable, have been reported in the literature: (i) reaction of dialkyl phosphites with sulfenyl chlorides,[13] sulfenyl cyanides,[14] thiosulfonates,[15–16] disulfides,[17] and sulfur, [18–21] (ii) condensation of phosphorchloridate with thiols [22–26] and (iii) redox-type reactions of phosphorus triesters with thiols in the presence of tellurium (IV) chloride. [27–28] However, all of these methods have problems, including drastic reaction conditions and also some severe side reactions. Surface-mediated solid phase reactions are of growing interest [29–35] because of their ease of set-up, work-up, mild reaction conditions, rate of the reaction, selectivity, high yields, lack of solvent and the low cost of the reactions in comparison with their homogeneous counterparts. The application of microwave energy to accelerate organic reactions is of increasing interest and offers several advantages over conventional techniques. [36] Synthesis of molecules that normally require long reaction times, can be achieved conveniently and very rapidly in a microwave oven. As a part of our efforts to explore the utility of surface-mediated reactions for the synthesis of organophosphorus compounds, [37–48] we report a new method for the preparation of phosphorothioates by reaction of diethyl phosphite with alkyl halides in the presence of a mixture of ammonium acetate/sulfur/alumina under solvent-free conditions using microwave irradiation which produces high yields of phosphorothioates (Scheme 1).

## Results and Discussion

**Scheme 2:**
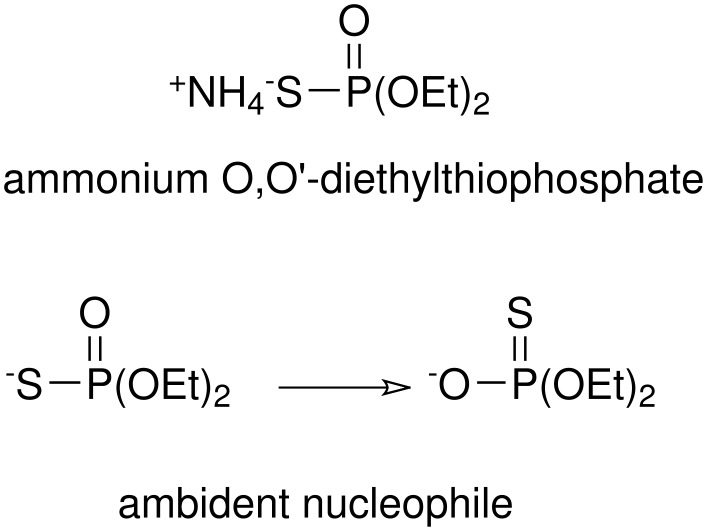
Ambident nucleophile ammonium *O,O'*-diethylthiophosphate

Recently we have found that ammonium *O,O*'-diethyl thiophosphate can be obtained by reaction of diethylphosphite in the presence of a mixture of ammonium acetate/sulfur/acidic alumina under solvent-free conditions using microwave irradiation. [49] This reagent can be used as an efficient reagent for the conversion of epoxides to thiiranes. This ambident nucleophile has two potentially attacking atoms (*S* or *O*) and can attack with either of them, depending on conditions, and mixtures are often obtained in the reaction with electrophilic centers (Scheme 2). [50]

We have found that the reaction of diethyl phosphite with alkyl halides in the presence of a mixture of ammonium acetate/sulfur/alumina under solvent-free conditions using microwave irradiation produces high yields of phosphothioates (*S*-alkylation, Scheme 1). [44] We decided to investigate the reaction of this ambident nuclophile under different conditions (different leaving groups and solvents).

**Scheme 3:**
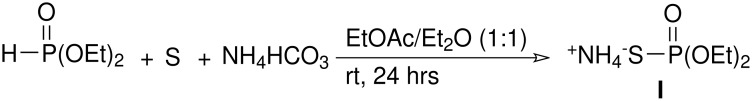
Synthesis of ammonium *O,O*'-diethyl thiophosphate

Firstly, we introduce a novel method for large-scale synthesis of ammonium *O,O*'-diethyl thiophosphate. The reaction of sulfur with diethylphosphite in the presence of ammonium hydrogen carbonate under reflux condition in a solvent mixture of ethyl acetate and diethyl ether (1:1) gave ammonium *O,O*'-diethyl thiophosphate in quantitative yield (Scheme 3).

**Scheme 4:**
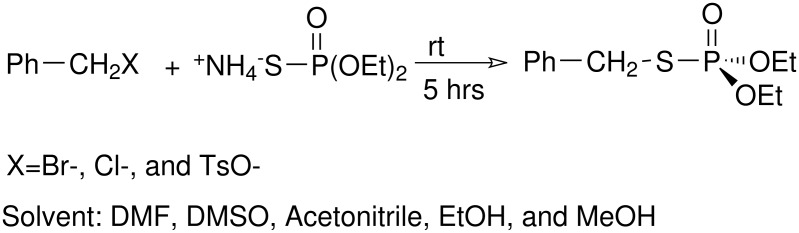
Solvent and leaving group effects on the synthesis phosphorothioates

The results of the reaction of this reagent with benzyl bromide, chloride and tosylate in different aporotic and protic solvents show that *S*-benzyl *O,O'*-diethyl phosphorothioate (*S*-alkylation) was formed as sole product (Scheme 4).

We conclude here that changing of leaving group and use of different media gives no *O*-alkylation product (i.e. changing from soft to hard leaving group and aprotic to protic solvent). Although ammonium *O,O*'-diethyl thiophosphate is a potential ambident nucleophile, only its soft center is reactive in this case.

**Scheme 5:**
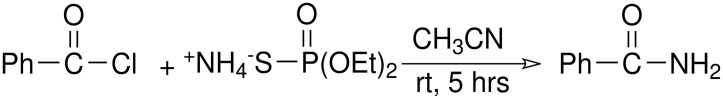
Reaction of ammonium *O,O*'-diethyl thiophosphate with benzoyl chloride

Recently the synthesis of *S*-thioacyl dithiophosphates has been reported as an efficient and chemoselective thioacylating agent using the reaction of acyl chlorides with dithiophosphoric acid in the presence of pyridine or triethylamine. [51–53] In another study we decided to investigate the reaction of the ambident nucleophile ammonium *O,O*'-diethyl thiophosphate salt with acyl chlorides. Reaction of ammonium *O,O*'-diethyl thiophosphate with benzoyl chloride, as a model compound, in acetonitrile gave benzamide as the major product (Scheme 5).

Benzoyl chloride reacts with ammonia (from ammonium *O,O*'-diethyl thiophosphate) faster than anion *O,O*'-diethyl thiophosphate to give benzamide. All efforts for solving this problem failed and in all cases benzamide was obtained as the major product.

**Scheme 6:**
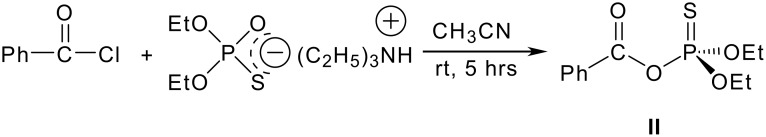
Reaction of triethylammonium *O,O*'-diethyl thiophosphate with benzoyl chloride

We decided to replace this ammonium ion with a triethyl ammonium ion and then to study the reaction of new salt with benzoyl chloride. Triethylammonium *O,O*'-diethyl thiophosphate was obtained by reaction of diethylphosphite, sulfur and triethyl amine. [54–57] We found that reaction of triethylammonium *O,O*'-diethyl thiophosphate with benzoyl chloride gave benzoyl *O,O*'-diethyl phosphorothioate with *O*-acylation product (Scheme 6).

We conclude that replacement of benzyl with benzoyl group (hard electrophilic center) gives the *O*-acylation product.

**Scheme 7:**
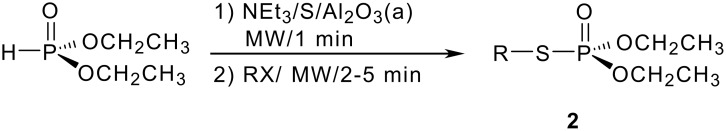
Synthesis of phosphorothioates using triethylammonium *O,O*'-diethyl thiophosphate using microwave irradiation.

As a part of our efforts to explore the utility of surface-mediated reactions for the synthesis of organophosphorus compounds, [16–18] herein we report a new method for the preparation of phosphorothioates by reaction of diethyl phosphite with alkyl halides in the presence of a mixture of triethylamine/sulfur/alumina under solvent-free conditions using microwave irradiation. We found that a mixture of alumina, sulfur, diethylphosphite and triethylamine under microwave irradiation gave triethylammonium *O,O*'-diethyl thiophosphate that can be used for the synthesis of phosphorothioates under solvent free conditions (Scheme 7, Table 1). As shown in Table 1, a wide range of alkyl halides in the presence triethylamine/sulfur/alumina reacted with diethyl phosphite, giving the required products **2** in moderate to good yields.

**Table 1:** Reaction of alkyl halides and tosylates in the presence of a mixture of triethyl amine/sulfure/alumina with diethylphosphite under solvent-free conditions

**2**	R	X	Reaction Time (min)	Yield %^a^

**a**	PhCH_2_	Br	3	62
**a**	PhCH_2_	OTs	3	67
**b**	PhCH_2_CH_2_	Br	2	72
**c**	*p*-NO_2_C_6_H_4_CH_2_	Br	4	70
**c**	*p*-NO_2_C_6_H_4_CH_2_	OTs	5	83
**d**	*o*-MeC_6_H_4_CH_2_	Br	3	65
**d**	*o*-MeC_6_H_4_CH_2_	Cl	3	65
**e**	*p*-ClC_6_H_4_CH_2_	OTs	2	67
**f**	*m*-ClC_6_H_4_CH_2_	OTs	2	55
**g**	*p*-MeC_6_H_4_CH_2_	Br	4	62
**h**	1-Butyl	Br	2	76
**I**	1-Hexyl	Cl	5	75

a: Isolated Yields

In summary, a simple work-up, low consumption of solvent, fast reaction rates, mild reaction conditions, good to excellent yields, relatively clean reactions with no tar formation make these methods an attractive and a useful contribution to present methods for the preparation of phosphorothioates. Studies on the reaction of ambident nucleophile ammonium *O,O*'-diethyl thiophosphate with benzyl halides and tosylate in different solvents show that only *S*-alkylation will be obtained as sole product. Reaction of this ambident nucleophile with benzoyl chloride (hard electrophilic center), gave the *O*-acylation product.

## Supporting Information

File 1 The additional file contains full experimental details
