# Supplementary material for: Synthesis of phosphorothioates using thiophosphate salts
Source: Beilstein J Org Chem. 2006 Mar 16;2:4. doi: 10.1186/1860-5397-2-4 (PMC1421414; doi:10.1186/1860-5397-2-4)
Supplement: File 1 — The additional file contains full experimental details [file Beilstein_J_Org_Chem-02-04-s001.doc]

**Experimental Section**

**General**

All chemicals were commercial products and distilled or recrystallized before use. All melting points were obtained by a Buchi 510 and are uncorrected. A commercially available pulse microwave at 2450 MHz (600 W) was used in all experiments. The infrared (IR) spectra were determined using a FT-IR Brucker-Vector 22. NMR spectra were taken with a 250 Brucker Avance instrument with the chemical shifts being reported as δ ppm and couplings expressed in Hertz. Silica gel column chromatography was carried out with Silica gel 100 (Merck No. 10184). Merck Silica-gel 60 F254 plates (No. 5744) were used for the preparative TLC.

**Synthesis of Ammonium *O,O*’-diethylthiophosphate [21] Using of a Mixture of Ammonium Hydrogen Carbonate, Sulfur and Diethylphosphite:**

Ammonium hydrogen carbonate (0.1 mol, 7.9 g) was added to a mixture of sulfur (0.1 mol, 3.2 gr) and diethylphosphite (0.1 mol, 13.8 g) in EtOAc/Et_2_O (300 mL, 1:1). Reaction mixture was stirred for 24 hrs at room temperature. Evaporation of the solvent under reduced pressure gave the pure ammonium *O,O*’-diethylthiophosphate as solid in quantitatively yield that can be recrystallized in ethanol.

**Ammonium *O,O’*-Diethyl Phosphorothioate (I).**

White crystals (Ethanol); m.p 122-125 °C; ^1^H-NMR (D_2_O/TMS-250 MHz): 1.21 (6H, t, *J*=7.1 Hz), 3.91-4.03 (4H, m); ^31^P-NMR (D_2_O/H_3_PO_4_-101.2 MHz): 55.14 ppm; Anal. Calcd for C_4_H_14_NPSO_3_. C, 25.67; H, 7.49; N, 7.49. Found: C, 25.51; H, 7.32; N, 7.20

**General Procedure for the Studies of Reaction of Ammonium *O,O’*-Diethyl Phosphorothioate (I)** **with Benzyl chloride, Bromide and Tosylate in Different Solvents at Room Temperature:**

Ammonium *O,O’*-diethyl phosphorothioate (5 mmol, 0.935 g) was dissolved in solvent. Benzyl bromide, chloride or tosylate (5 mmol) was added to reaction mixture and stirred for 5 hrs. Evaporation of solvent and washing with sodium hydrogen carbonate (50x3 mL) to give *S*-benzyl *O,O’*-diethyl phosphorothioate as sole product. Product was monitored with ^31^P-NMR for determination of products ratio.

**Study of the Reaction of Triethyl Ammonium *O,O’*-Diethyl Phosphorothioate with Benzoyl Chloride in Acetonitrile at Room Temperature:**

Triethyl ammonium *O,O’*-diethyl phosphorothioate (5 mmol, 1.35 g) [22] was dissolved in acetonitrle (20 mL). Benzoyl chloride was added to reaction mixture and stirred for 5 hrs at room temperature. Evaporation of solvent to give *S*-benzoyl *O,O’*-diethyl phosphorothioate as major product. Products ratio was determined by monitoring of ^31^P-NMR.

**Triethylmmonium *O,O’*-Diethyl Phosphorothioate:**

Colorless oil; ^1^H-NMR (CDCl_3_/TMS-250 MHz): 1.01-1.22 (15H, m ), 2.94-3.10 (6H, m), 3.71-4.03 (4H, m); ^13^C-NMR: 8.42, 15.9 (d, J_pc_=8.0 Hz), 45.8, 62.1 (d, J_pc_=5.7 Hz); ^31^P-NMR (CDCl_3_/H_3_PO_4_-101.2 MHz): 59.88 ppm; Anal. Calcd for C_10_H_26_NPSO_3_. C, 44.28; H, 5.59; N, 5.17. Found: C, 44.10; H, 5.39; N, 5.02.

***S*-Benzoyl *O,O’*-Diethyl Phosphorothioate (II)**

Colorless oil; ^1^H-NMR (CDCl_3_/TMS-250 MHz): 1.42 (6H, dt, *J*=8.0, J=1.0 Hz), 4.31-4.45 (4H, m), 7.43-7.7.69 (3H, m), 8.01-8.09 (2H, m); ^31^P-NMR (CDCl_3_/H_3_PO_4_-85%): 60.38 ppm; ^13^C-NMR: 15.7 (d, J_pc_=7.6 Hz), 65.7 (d, J_pc_=5.6 Hz), 128.4, 128.7, 130.6, 134.42, 161.2 (d, 7.8 Hz); IR (neat): 1744 (C=O), 1162(P-O-Et), 694 (P=S) cm^-1^; Anal. Calcd for C_11_H_15_PSO_4_. C, 48.17; H, 5.47. Found: C, 47.89; H, 5.53.

**General Procedure for the Synthesis of Phosphorothioates Using of a One-Pot Reaction of Alkyl Halides with the Mixture of Diethyl Phosphite in the Presence of Triethyl Amine/Sulfur/Alumina under Solvent –Free Conditions:**

This solvent-free reaction method is operationally simple. 10 mmol of the reagent was prepared by the combination of triethyl amine (10 mmol, 1.01 g), sulfur (10 mmol, 0.32 g) and alumina (Al_2_O_3_, acidic, 2.5 g). Diethyl phosphite (10 mmol, 1.38 g) was added to this mixture and the mixture was irradiated by microwave for 1 min using 720 W. The alkyl halide (9 mmol) was added to this reagent (solid alkyl halides need to be grinded) and was irradiated by microwave for 2-5 min using 720 W (A kitchen-type microwave was used in all experiments). Chromatography through a plug of silica gel with EtOAc/*n*-hexane (1:9 to 5:5) and evaporation of the solvent under reduced pressure gave the pure products as oils in 55-83% yields.

***S*-Benzyl *O,O’*-Diethyl Phosphorothioate (2a)**

Colorless oil; [21, 44] ^1^H-NMR (CDCl_3_/TMS-250 MHz): 1.31 (6H, t, *J*=7.1 Hz), 4.01-4.25 (6H, m), 7.27-7.29 (5H, m); ^31^P-NMR (CDCl_3_/H_3_PO_4_-85%): 27.05 ppm; ^13^C-NMR: 15.6 (d, J_PC_=7.2 Hz), 34.5 (d, J_PC_=3.9 Hz), 63.1 (d, J_PC_=5.8 Hz), 127.3, 128.3, 128.6, 137.4 (d, J_PC_=4.9 Hz); IR (neat): 1260 (P=O), 1162-1010(P-O-Et) cm^-1^; Anal. Calcd for C_11_H_17_PSO_3_. C, 50.77; H, 6.54. Found: C, 50.62; H, 6.45.

***O,O’*-Diethyl *S*-(2-phenyl)ethyl Phosphorothioate (2b)**

Colorless oil; [44] ^1^H-NMR (CDCl_3_/TMS-250 MHz): 1.35 (6H, t, *J*=7.1 Hz), 2.93-3.12 (2H, m), 4.01-4.23 (4H, m), 7.15-7.35 (5H, m); ^31^P-NMR (CDCl_3_/H_3_PO_4_-85%): 27.92 ppm; ^13^C-NMR (CDCl_3_/TMS-62.9 MHz): 15.6 (d, J_PC_=7.3 Hz), 31.9 (d, J_PC_=3.8 Hz), 37.1 (d, J_PC_=5.2 Hz), 63.4 (d, J_PC_=6.1 Hz), 126.6, 128.4, 128.5, 139.3; IR (neat): 1255 (P=O), 1155-1011(P-O-Et) cm^-1^; Anal. Calcd for C_12_H_19_PSO_3_. C, 52.55; H, 6.93. Found: C, 52.36; H, 6.76.

***O,O’*-Diethyl *S*-(*p*-Nitrophenyl)methyl Phosphorothioate (2c)**

Colorless oil; [44] ^1^H-NMR (CDCl_3_/TMS-250 MHz): 1.19 (6H, t, *J*=7.1 Hz), 3.84-4.06 (6H, m), 7.46 (2H, d, *J*=8.7 Hz), 8.05 (2H, d, *J*=8.7 Hz);^31^P-NMR (CDCl_3_/H_3_PO_4_-101.2MHz): 25.64 ppm; ^13^C-NMR (CDCl_3_/TMS-62.9 MHz): 15.4 (d, J_PC_=7.0 Hz), 33.5 (d, J_PC_=3.8 Hz), 63.4 (d, J_PC_=6.0 Hz), 123.3, 129.6, 145.4 (d, J_PC_=3.9 Hz), 146.7; IR (neat): 1254 (P=O), 1100-970 (P-O-Et) cm^-1^; Anal. Calcd for C_11_H_16_NPSO_5_. C, 43.27; H, 5.24; N, 4.59. Found: C, 43.42; H, 5.32; N, 4.36.

***O,O’*-Diethyl *S*-(*o*-Methylphenyl)methyl Phosphorothioate (2d)**

Colorless oil; [44] ^1^H-NMR (CDCl_3_/TMS-250 MHz): 0.89 (6H, t, *J*=7.1 Hz), 2.03 (3H, s), 3.56-3.70 (6H, m), 6.71-7.20 (4H, m);^31^P-NMR (CDCl_3_/H_3_PO_4_-101.2 MHz): 26.24 ppm; ^13^C-NMR (CDCl_3_/TMS-62.9 MHz): 15.6 (d, J_PC_=7.3 Hz), 18.7, 32.7 (d, J_PC_=3.8 Hz), 63.0 (d, J_PC_=6.1 Hz), 125.9, 127.7, 129.7, 130.2, 134.9 (d, J_PC_=5.9 Hz), 136.3; IR (neat): 1246 (P=O), 1100-960 (P-O-Et) cm^-1^; Anal. Calcd for C_12_H_19_PSO_3_. C, 52.55; H, 6.93. Found: C, 52.40; H, 6.83.

***O,O’*-Diethyl *S*-(*p*-Chlorophenyl)methyl Phosphorothioate (2e)**

Colorless oil; ^1^H-NMR (CDCl_3_/TMS-250 MHz): 0.99 (6H, t, *J*=7.0 Hz), 3.61-3.96 (6H, m), 6.90-7.18 (4H, m); ^31^P-NMR (CDCl_3_/H_3_PO_4_-101.2 MHz): 26.27 ppm; ^13^C-NMR (CDCl_3_/TMS-62.9 MHz): 15.7 (d, J_PC_=8.0 Hz), 33.9 (d, J_PC_=3.3 Hz), 63.3 (d, J_PC_=5.8Hz), 128.5, 130.1, 133.1, 136.1 (d, J_PC_=4.7 Hz);IR (neat): 1235 (P=O), 1130-950 (P-O-Et) cm^-1^; Anal. Calcd for C_11_H_16_ClPSO_3_. C, 44.82; H, 5.43. Found: C, 45.02; H, 5.32.

***O,O’*-Diethyl *S*-(*m*-Chlorophenyl)methyl Phosphorothioate (2f)**

Colorless oil; ^1^H-NMR (CDCl_3_/TMS-250 MHz): 1.28 (6H, t, *J*=7.1 Hz), 3.92-4.19 (6H, m), 7.21-7.29 (3H, m), 7.35 (1H, s); ^31^P-NMR (CDCl_3_/H_3_PO_4_-101.2 MHz): 26.31 ppm; ^13^C-NMR (CDCl_3_/TMS-62.9 MHz): 15.9 (d, J_PC_=7.3 Hz), 34.3 (d, J_PC_=3.9 Hz), 63.7 (d, J_PC_=5.9 Hz), 127.1, 127.8, 129.0, 129.9, 134.4, 139.6 (d, J_PC_=4.5 Hz); IR (neat): 1255 (P=O), 1140-950 (P-O-Et) cm^-1^; Anal. Calcd for C_11_H_16_ClPSO_3_. C, 44.82; H, 5.43. Found: C, 44.95; H, 5.22.

***O,O’*-Diethyl *S*-(*p*-Methylphenyl)methyl Phosphorothioate (2g)**

Colorless oil; [44] ^1^H-NMR (CDCl_3_/TMS-250 MHz): 1.21 (6H, t, *J*=7.2 Hz), 2.24 (3H, s), 3.92 (2H, d, 13.5 Hz), 3.86-4.13 (4H, m), 7.03 (2H, d, *J*=7.7 Hz), 7.16 (2H, d, *J*=7.7 Hz); ^31^P-NMR (CDCl_3_/H_3_PO_4_-101.2 MHz): 26.92 ppm; ^13^C-NMR (CDCl_3_/TMS-62.9 MHz): 15.8 (d, J_PC_=7.2 Hz), 20.9, 34.6 (d, J_PC_=3.6 Hz), 63.3 (d, J_PC_=5.7 Hz), 128.6, 129.1, 134.3 (d, J_PC_=5.5 Hz), 137.1; IR (neat): 1250 (P=O), 1140-960 (P-O-Et) cm^-1^; Anal. Calcd for C_12_H_19_PSO_3_. C, 52.55; H, 6.93. Found: C, 52.31; H, 6.76.

***S*-(1-Buthyl) *O,O*-Diethyl Phosphorodithioate (2h)**

Colorless oil; [21] ^1^H-NMR (CDCl_3_/TMS-250 MHz): 0.46 (3H, t, *J*=7.2 Hz), 0.8-1.10 (8H, m), 1.17 (2H, qui, 8.2 Hz), 2.25-2.45 (2H, m); 3.55-3.82 (4H, m); ^31^P-NMR (CDCl_3_/H_3_PO_4_-101.2 MHz): 27.66 ppm; IR (neat): 1256 (P=O), 1120-960 (P-O-Et) cm^-1^; Anal. Calcd for C_8_H_20_PSO_3_. C, 42.29; H, 8.81. Found: C, 42.49; H, 8.85.

***O,O’*-Diethyl *S*-(1-Hexyl) Phosphorothioate (2i)**

Colorless oil; ^1^H-NMR (CDCl_3_/TMS-250 MHz): 0.87 (3H, t, *J*=7.0 Hz), 1.20-1.43 (12H, m), 1.64 (2H, qui, 7.5 Hz), 2.75-2.95 (2H, m); 4.05-4.35 (4H, m); ^31^P-NMR (CDCl_3_/H_3_PO_4_-101.2 MHz): 28.58 ppm; ^13^C-NMR (CDCl_3_/TMS-62.9 MHz): 13.9, 15.9 (d, J_PC_=7.2 Hz), 22.4, 28.2, 30.7 (d, J_PC_=5.8 Hz), 30.9 (d, J_PC_=3.9 Hz), 31.2, 63.4 (d, J_PC_=6.0 Hz); IR (neat): 1256 (P=O), 1120-960 (P-O-Et) cm^-1^; Anal. Calcd for C_10_H_24_PSO_3_. C, 47.06; H, 9.41. Found: C, 46.85; H, 9.30.
